# *Ex Vivo* Model Unravelling Cell Distribution Effect in Hydrogels for Cartilage Repair

**DOI:** 10.14573/altex.1704171

**Published:** 2017-09-08

**Authors:** Vivian H. M. Mouser, Noël M. M. Dautzenberg, Riccardo Levato, Mattie H. P. van Rijen, Wouter J. A. Dhert, Jos Malda, Debby Gawlitta

**Affiliations:** 1 Department of Orthopaedics, University Medical Center Utrecht, Utrecht University, Utrecht, The Netherlands; 2 Department of Equine Sciences, Faculty of Veterinary Medicine, Utrecht University, Utrecht, The Netherlands; 3 Department of Oral and Maxillofacial Surgery & Special Dental Care, University Medical Center Utrecht, Utrecht University, Utrecht, The Netherlands

**Keywords:** cartilage repair, regeneration, osteochondral plug, GelMA/gellan gum

## Abstract

The implantation of chondrocyte-laden hydrogels is a promising cartilage repair strategy. Chondrocytes can be spatially positioned in hydrogels and thus in defects, while current clinical cell therapies introduce chondrocytes in the defect depth. The main aim of this study was to evaluate the effect of spatial chondrocyte distribution on the reparative process. To reduce animal experiments, an *ex vivo* osteochondral plug model was used and evaluated. The role of the delivered and endogenous cells in the repair process was investigated.

Full thickness cartilage defects were created in equine osteochondral plugs. Defects were filled with (A) chondrocytes at the bottom of the defect, covered with a cell-free hydrogel, (B) chondrocytes homogeneously encapsulated in a hydrogel, and (C, D) combinations of A and B with different cell densities. Plugs were cultured for up to 57 days, after which the cartilage and repair tissues were characterized and compared to baseline samples. Additionally, at day 21, the origin of cells in the repair tissue was evaluated.

Best outcomes were obtained with conditions C and D, which resulted in well-integrated cartilage-like tissue that completely filled the defect, regardless of the initial cell density. A critical role of the spatial chondrocyte distribution in the repair process was observed. Moreover, the osteochondral plugs stimulated cartilage formation in the hydrogels when cultured in the defects. The resulting repair tissue originated from the delivered cells.

These findings confirm the potential of the osteochondral plug model for the optimization of the composition of cartilage implants and for studying repair mechanisms.

## 1 Introduction

The implantation of tissue-engineered constructs is a promising approach for the treatment of articular cartilage defects. Articular cartilage is a thin connective tissue, lining the ends of long bones, and consisting of predominantly water, aggrecan, collagen type II, and chondrocytes (Almarza and Athanasiou, 2004; Browne and Branch, 2000). The tissue can be roughly divided into three zones: the superficial zone, intermediate or middle, and the deep zone, which is attached to the bone via the calcified layer. In each zone, the ratio, organization, and orientation of the matrix components is different, as well as the chondrocyte density, organization, and their protein expression (Klein et al., 2009; Schuurman et al., 2015). As adult articular cartilage lacks vascularization and innervation, and contains relatively low total cell numbers; the tissue has a limited regenerative capacity. As a result, untreated cartilage injuries generally lead to osteoarthritic changes in the joint (Browne and Branch, 2000; Prakash and Learmonth, 2002).

The most commonly used strategies to repair chondral defects are based on marrow stimulation techniques (microfracture) or on active cell delivery (autologous chondrocyte implantation, ACI) (Browne and Branch, 2000; Judet et al., 2005; Mandelbaum et al., 2007). Third-generation ACI-based strategies involve chondrocyte seeding into a biomaterial or scaffold, such as collagen type I/III membranes, that is fixated in the defect with, e.g., fibrin glue (Brittberg, 2010; Marlovits et al., 2006). This matrix-induced ACI (MACI) technique was demonstrated to stimulate the formation of hyaline-like repair tissue (Ebert et al., 2017; Meyerkort et al., 2014). However, full restoration of the defect site or reconstruction of the complex depth-dependent architecture of native cartilage is not achieved, indicating the need for further development.

Novel strategies for cell delivery and cartilage tissue engineering employ cell-laden hydrogels, which can be cast or three-dimensionally (3D) bioprinted with depth-dependent architectures and/or patient-specific geometries (Mouser et al. 2016a). Subsequently, the constructs can be pre-cultured *in vitro* to stimulate chondrocyte differentiation before implantation. A promising hydrogel for the biofabrication of such implants is gelatin methacryloyl (gelMA) with gellan gum, which can be UV cross-linked, retains its shape during crosslinking and culture, and was demonstrated to support chondrogenesis of embedded chondrocytes (Mouser et al., 2016b). Additionally, gelMA/gellan is compatible with 3D printing techniques, which facilitate accurate positioning of the (cell-laden) biomaterial (Mouser et al., 2016b; Melchels et al., 2014).

For clinical translation of new cartilage regeneration technologies, evaluation of the approaches in animal models is indispensable. However, in view of the reduction and replacement of animal studies, an *ex vivo* osteochondral (OC) plug model was developed (de Vries-van Melle et al., 2012, 2014a, b). This model entails OC plugs obtained from cadaveric joints, which can be cultured in a recently developed culture platform (Schwab et al., 2016). A cartilage defect of the desired depth can be generated within the cartilage tissue of the OC plug. Accordingly, cartilage repair mechanisms mimicking different treatment strategies may be studied in this model (de Vries-van Melle et al., 2012). Therefore, this model may be used to reduce the need for animal experiments while developing and optimizing hydrogel constructs for cartilage repair.

An important aspect for the clinical translation of new cartilage regenerative therapies, e.g., cell-laden hydrogels, is to understand the repair mechanisms underlying the new therapy. Current clinical therapies are based on the introduction of a dense cell layer in the defect depth, e.g., by means of microfracture or application of ACI (Huang et al., 2016). Although the initial situation is different with MACI, as the chondrocytes are pre-seeded in the collagen membrane, it was observed that the seeded chondrocytes migrate from the patch into the fibrin glue in the defect depth during the first days after implantation (Jones et al., 2008). Therefore, it has been suggested that cartilage repair in MACI also originates from a dense chondrocyte layer in the defect depth (Brittberg et al., 2001; Nixon et al., 2015). Contrarily, new hydrogel-based repair strategies allow homogeneous chondrocyte encapsulation and delivery into the defect, representing an alternative chondrocyte delivery approach. In order to optimize hydrogel-based therapies for clinical use, it is crucial to better understand the associated repair mechanisms (Huang et al., 2016) and to optimize the spatial chondrocyte distribution for cartilage repair. This study aimed to investigate the effect of spatial variations in chondrocyte distribution within a hydrogel construct on cartilage repair and to investigate the effect of the *ex vivo* OC plug model on tissue formation within the hydrogel, as well as to assess the contribution of endogenous and delivered cells to the formation of repair tissue.

## 2 Materials and methods

### Experimental design

**Fig. 1:**
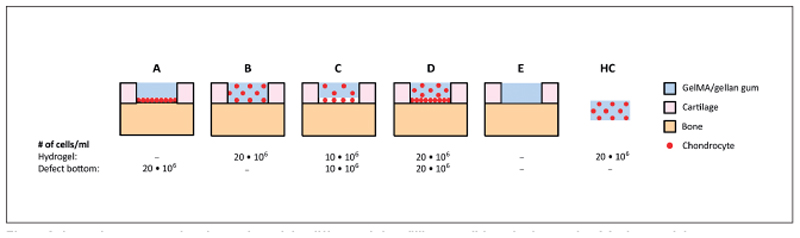
Schematic cross-sectional overview of the different defect filling conditions in the *ex vivo* OC plug model Chondrocytes were seeded at the bottom of the defect (A, 20x10^6^ cells/ml), homogeneously in the hydrogel (B, 20x10^6^ cell/ml), both at the bottom of the defect and in the hydrogel (C, 10x10^6^ cell/ml + 10x10^6^ cell/ml, respectively; D, 20x10^6^ cell/ml + 20x10^6^ cell/ml, respectively). An empty hydrogel was used as control (E). All defects were filled with a total of 10 μl hydrogel. To evaluate the influence of the *ex vivo* OC plug model on the tissue production of the delivered chondrocytes, a cell-laden hydrogel control (HC, 20x10^6^ cell/ml) also was cultured.

Equine OC plugs were isolated and full-thickness cartilage defects were created. Defects were filled with gelMA/gellan using four different spatial chondrocyte distributions (20x10^6^/ml of the defect volume; Fig. 1). Condition A consisted of allogenic chondrocytes seeded at the bottom of the defect that were covered with an empty hydrogel. In condition B, the allogenic chondrocytes were homogeneously encapsulated in the hydrogel. Conditions C and D were a combination of conditions A and B, in which chondrocytes were both seeded at the defect bottom and homogeneously encapsulated in the hydrogel. In condition D the cell concentration was double that of condition C. Cartilage defects were filled with an empty hydrogel in condition E. Additionally, to investigate if the *ex vivo* OC plug model influences matrix-production of implanted chondrocytes, chondrocyte-laden hydrogel constructs (20x10^6^/ml) were molded and cultured floating in medium as a control. Plugs from all conditions were harvested at days 0 or 1 (baseline), 29, and 57 to evaluate the native cartilage and cartilage tissue formation in the defect area. In a separate experiment, plugs filled with condition D were collected at day 21 to compare DNA profiles of the newly formed tissue to those of the OC plug and chondrocyte donors.

### Chondrocytes

Primary chondrocytes were harvested from full thickness cartilage of metacarpophalangeal joints of a fresh equine cadaver obtained from the local slaughterhouse (N = 1, 5 years old). After confirming that the cartilage was macroscopically healthy, it was cut into small pieces that were digested overnight at 37°C in Dulbecco’s Modified Eagle Medium (DMEM, 31885, Gibco), supplemented with collagenase II (1.5 μg/ml, Worthington Biochemical Corp, Vollenhove, The Netherlands), hyaluronidase (1 mg/ml, Sigma Aldrich), fetal bovine serum (FBS, 10% v/v, Gibco) and pen/strep (1%, final concentration: 100 units/ml penicillin and 100 μg/ml streptomycin, Gibco). After digestion, the suspension was filtered through a 40 μm cell strainer and the chondrocytes were stored at passage 0 in liquid nitrogen until further use.

Before use, the chondrocytes were expanded in monolayer culture for 14 days (seeding density of 5x10^3^ cells/cm^2^, ~3.4 population doublings as the chondrocytes required a few days to reenter the cell cycle after being frozen) in DMEM (31885, Gibco) supplemented with FBS (10% v/v) and pen/strep (1%). The chondrocytes were harvested at passage 1 when they reached 80-90% confluency to be used for the experiments.

### Osteochondral plugs

Equine metacarpophalangeal joints were obtained from the local slaughterhouse (N = 1, 3 years old for the evaluation of tissue repair; N = 1, 5 years old for DNA profiling). OC plugs (diameter 8.5 mm, bone height 3 mm, cartilage thickness~1 mm) were drilled and cut under sterile conditions using a surgical trephine burr (Smith and Nephew, Hoofddorp, The Netherlands). Next, full thickness cartilage defects were created with a 4 mm biopsy punch (Stiefel, Brentford, UK). The edges and bottom of the defect were cleaned with a sharp spoon (MF Dental, Mantel, Germany) while maintaining the calcified layer as much as possible. Prepared plugs were incubated overnight in incubation medium (α-Minimum Essential Medium (α-MEM, 22561 Gibco) supplemented with FBS (10% v/v), 2x concentrated pen/strep (final concentration: 200 units/ml penicillin and 200 μg/ml streptomycin, Gibco), and Gentamicin sulphate (final concentration 50 μg/ml, Lonza BioWhittaker, Breda, The Netherlands)).

The following day, the plugs were inserted into a culture platform, which provided separate medium compartments for the cartilage and bone tissue (LifeTec Group B.V., Eindhoven, The Netherlands) (Schwab et al., 2016). The required numbers of chondrocytes defined for conditions A, C, and D (Fig. 1) were seeded by pipetting 10 μl cell-suspension into the defect. Plugs were incubated in the culture platform for 2 hours at 37°C in a humidified environment to allow chondrocytes to attach to the defect bottom. During this time, the bone compartment was supplemented with bone medium (α-MEM (22561 Gibco, with 2 mM l-glutamax), FBS (10%), L-ascorbic acid-2-phosphate (0.2 mM, Sigma Aldrich), pen/strep (1%), β-glycerophosphate (10 mM, Sigma Aldrich) and dexamethasone (10 nM, Sigma Aldrich)). Next, incubation medium was carefully pipetted onto the cartilage until completely covered, and plugs were incubated for another hour after which the medium was removed from the cartilage and defect before continuing with the hydrogel filling.

To fill the defects, gelMA was synthesized from porcine gelatin and 10% gelMA + 0.5% gellan gum solution was freshly prepared, both as previously described (Mouser et al., 2016b; Melchels et al., 2014). GelMA/gellan (10 μl per defect) was dispensed into the defect without chondrocytes (conditions A, E) or with embedded chondrocytes (conditions B, C, D; Fig. 1). Filled defects were covered with silicon-coated microscopy cover glasses (Sigmacote, Sigma-Aldrich, Zwijndrecht, The Netherlands) and cross-linked with UV light (UV-Handleuchte lamp A, Hartenstein, Germany, wavelength: 365 nm, intensity: 1.2 mW/cm^2^). Finally, cover glasses were removed and the OC plugs were cultured with bone medium in the bone compartment and chondrogenic differentiation medium (high glucose DMEM (D6429, Sigma Aldrich) supplemented with ITS + premix (1%, BD biosciences, Breda, The Netherlands), dexamethasone (0.1 μM, Sigma Aldrich), L-ascorbic acid-2-phosphate (0.2 mM, Sigma Aldrich), recombinant human transforming growth factor-β1 (TGF-β1, 10 ng/ml, Peprotech) and pen/strep (1%)) in the cartilage compartment. Hydrogel control (HC) samples were prepared by casting chondrocyte-laden gelMA/gellan (20x10^6^ cells/ml) in Teflon cylindrical molds (diameter: 4 mm, height: 2 mm). HCs were UV cross-linked and cultured in chondrogenic differentiation medium (1 ml medium per sample, 4 hydrogels per timepoint). Samples for baseline detection were obtained at day 0 for the native cartilage and day 1 for the defect filling (# plugs = 4, for both groups). The remaining samples were harvested at days 29 (# plugs = 3 for conditions A-D and HC, # plugs = 2 for condition E), 21 (# plugs = 4, only condition D), and 57 (# plugs = 4 for conditions A-D and HC, # plugs = 3 for condition E). Medium samples were taken from the cartilage compartment upon each medium change (twice a week).

### Quantitative biochemical analysis

At days 0 or 1, 29 and 57, the cartilage of the OC plugs was cut in half and the tissue inside the defect area was separated from the surrounding cartilage. Tissue samples were weighed (wet weight), freeze dried, and weighed again (dry weight) to determine the water content. Next, samples were digested overnight at 56°C in 200 μl papain digestion buffer (0.2 M NaH_2_PO_4_ + 0.01 M EDTA*2 H_2_O in milliQ, pH = 6.0) supplemented with 250 μl/ml papain solution (16-40 units/mg protein, Sigma Aldrich) and 0.01 M cysteine (Sigma Aldrich). In addition to tissue samples, medium samples were taken from the cartilage compartment upon each medium change. The amount of sulfated glycosaminoglycans (GAGs), both in the digested tissue and the medium, as a measure of proteoglycans, was determined with a dimethylmethylene blue (DMMB, pH = 3.0) assay (Farndale et al., 1982), using known concentrations of chondroitin sulfate C (Sigma Aldrich) as a reference. Quantification of DNA was performed with a Quant-iT PicoGreen dsDNA kit (Molecular Probes, Invitrogen, Carlsbad, USA) using a spectrofluorometer (Biorad, Veenendaal, The Netherlands) according to the instructions of the manufacturer.

### Histology and immunohistochemistry

After harvesting half of the cartilage and repair tissue, the remaining tissue was fixed overnight in formalin (10%) and decalcified for 21 days in ethylenediaminetetraacetic acid solution (EDTA, 12.5%, VWR chemicals, Amsterdam, The Netherlands) at 37°C. The OC plugs were embedded in paraffin and tissue sections (5 μm) were cut for the visualization of matrix components (Abbadessa et al., 2016). Safranin-O/Fast green staining was performed to visualize collagens (green/blue) and GAGs (red) (Wall and Board, 2014). To visualize the orientation of the collagen fibers, other sections were stained with picrosirius red (1 μg/ml, 60 min) with Weigert’s hematoxylin as counterstain. Collagen types I, II, and VI were visualized with immunohistochemistry as previously described (Abbadessa et al., 2016). The stained sections were scanned with a NanoZoomer-XR Digital slide scanner (C12000-02, Hamamatsu, Almere, The Netherlands) while the picrosirius stained sections were evaluated with a light microscope and a polarizing filter (BX51 with a DP70 camera, Olympus, Hamburg, Germany).

### Origin of repair tissue

At day 21, the newly formed tissue overgrowing the native cartilage was isolated separately from the defect filling. In order to manage this, the defect area was covered with a biopsy punch while pulling the tissue layer of the native cartilage surrounding the biopsy punch. Tissue samples, including the cartilage and chondrocyte samples taken at day 0, were washed in ice-cold PBS and digested overnight in digestion buffer (100 mM NaCl, 10 mM TrisCl pH 8, 25 mM EDTA pH 8, 0.5% sodium dodecyl sulfate, 0.1 mg/ml proteinase K from *Tritirachium album*, 50°C). DNA was extracted with phenol/chloroform/isoamyl alcohol and purified via precipitation with ammonium acetate (7.5M, ½x sample volume) and ethanol (100%, 2x sample volume). Purified DNA was rinsed with ethanol (70%), air dried, and resuspended in Tris-EDTA buffer (TE buffer, 10 mM Tris, 1 mM EDTA, pH 8). DNA profiles were generated and compared by Van Haeringen Laboratorium B.V. (Wageningen, The Netherlands).

### Statistical analyses

Statistical analysis was performed with SPSS software (version 21, IMB Corp.). A one-way ANOVA was used to determine differences between conditions (and the baseline values when analyzing the tissue surrounding the defect) at day 29 or day 57. Normality was assumed and homogeneity was determined with a Levene test. When the ANOVA was significant (p < 0.05) and sample groups were homogeneous, a Bonferroni post-hoc test was performed. When samples groups were not homogeneous a Games-Howel post-hoc test was used. In all graphs, the error bars depict the standard error of the mean (SEM).

## 3 Results

### 3.1 Evaluation of the osteochondral plugs

**Fig. 2:**
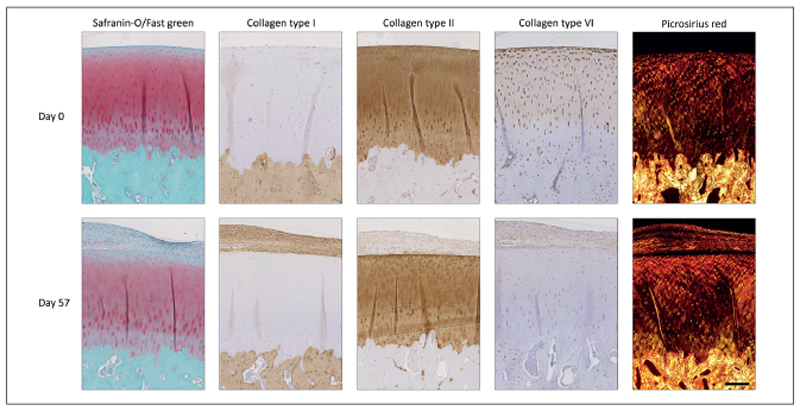
Change in native cartilage and bone morphology of the OC plugs during the culture period Images of day 57 are from the tissue surrounding the cartilage defect which was filled with condition A and are representative for all conditions which received chondrocytes in the defect (# plugs = 16). GAG content of the cartilage stained less intensely after 57 days of culture. Additionally, new tissue grew over the defect area and covered the cartilage surface during culture. Scale bar represents 400 μm and is valid for all images.

The overall morphology of the bone and native cartilage surrounding the defect area of the plugs was similar after 57 days of culture to the morphology of day 1 samples, as observed in the safranin-O staining (Fig. 2). In the native cartilage, the intensity of the safranin-O staining decreased during the culture period for all plugs. Nevertheless, the native cartilage stained positive for collagen type II and negative for collagen type I at day 57, with similar intensity to what was observed at day 0. The orientation of the collagen fibers remained unchanged during culture and the cells kept their pericellular matrix intact as visualized with picrosirius red and collagen type VI staining. However, the collagen type VI staining intensity within the extracellular matrix decreased during culture.

Unexpectedly, the formation of an additional tissue layer was observed on top of the superficial cartilage of the plugs, which had defects filled with cells, regardless of the delivery method (Fig. 2). Generally, this newly formed tissue stained positive for collagen type I, and to a lesser extent for collagen type II, while only sporadic safranin-O positive areas were observed. It contained a relatively high cell density and cells had a stretched morphology. Collagen type VI was found in the extracellular matrix of the newly formed tissue, but only sparsely in the pericellular matrix of the present cells. Additionally, picrosirius red staining revealed a collagen fiber orientation parallel to the cartilage surface in this tissue layer.

**Fig. 3:**
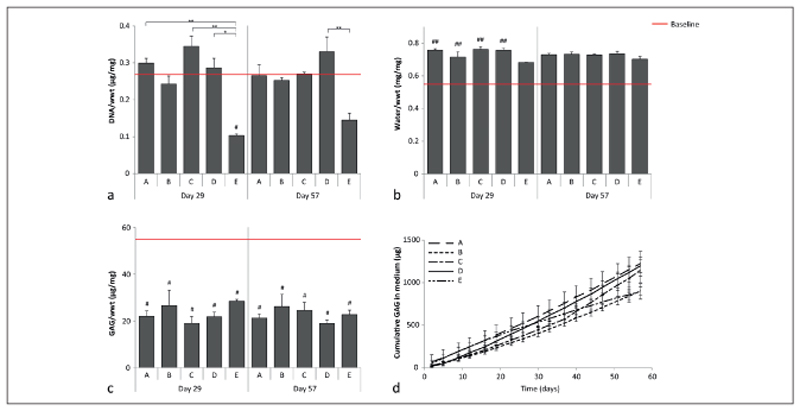
Quantitative measurement of DNA, GAGs and water content of the native cartilage surrounding the defects filled with the different conditions a) DNA normalized to wwt; b) Water content normalized to wwt; c) GAG normalized to wwt; d) cumulative GAGs measured in the medium. Baseline (red) indicates the average value measured at day 0 (# plugs = 3). For conditions A-D, 3 and 4 plugs were measured at days 29 and 57, respectively, while 2 and 3 plugs were measured respectively for E. # represents a significant difference compared to the baseline (#, p < 0.05; ##, p < 0.01), and * indicates a significant difference between the connected conditions (*, p < 0.05; **, p < 0.01).

Quantitative measurements of the native cartilage surrounding the defects showed minor changes compared to baseline values (day 0). DNA content normalized to the sample wet weight (wwt) decreased at day 29 compared to the baseline for condition E, while no differences compared to the baseline were measured at day 57 (Fig. 3a). The water content normalized to the wwt was significantly increased at both time points compared to the baseline, except for condition E (Fig. 3b). GAG per wwt was decreased for all conditions at day 29 and 57 compared to the baseline (Fig. 3c). Additionally, a constant GAG release was measured in the medium for all conditions (Fig. 3d). On average, 19.1 ±5.2 μg GAG leached into the medium per day.

### 3.2 Cartilage regeneration in the defect

**Fig. 4:**
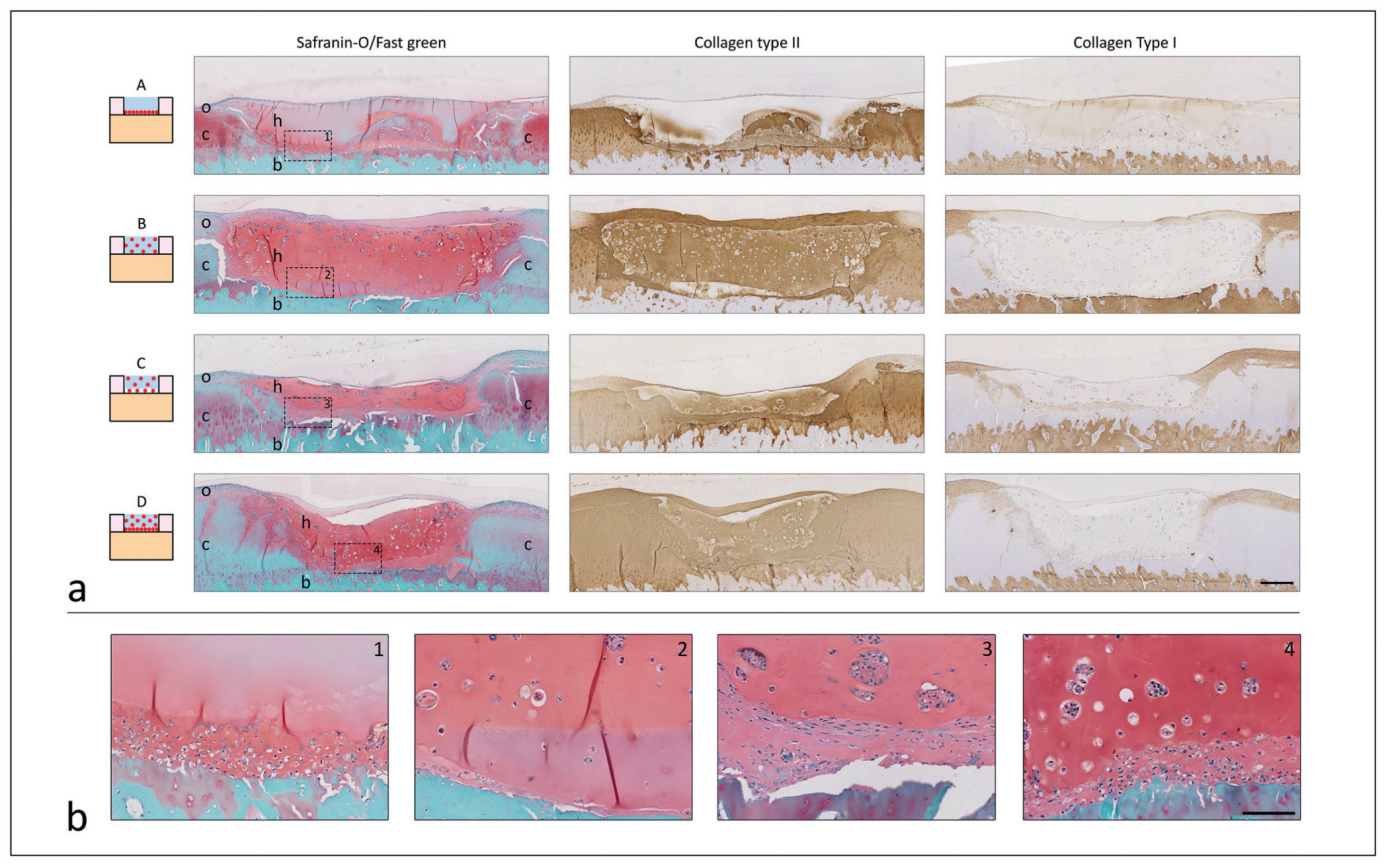
Histological analysis of cartilage defects filled with conditions A-D at day 57 a) Cross-sectional overview of each condition (# plugs = 4). Scale bar represents 400 μm and is the same for all histological images. In the safranin-O images, c, native cartilage; b, bone; h, hydrogel; o, tissue outgrowth. b) Magnification of the area indicated with the dotted square and number in the safranin-O pictures of the cross-sectional overview (a). From left to right conditions A-D. Scale bar represents 100 μm and is the same for all enlarged images.

Multiple differences in repair tissue were observed between the tested conditions (Fig. 4a). In plugs of condition A, a dense tissue layer was visible at days 29 and 57 at the defect bottom and at the cartilage-hydrogel interface. This tissue layer contained relatively high cell numbers and stained positive for collagen type II and safranin-O and negative for collagen type I. Additionally, the tissue layer filled irregularities at the sides and near the tidemark (Fig. 4a,b; for more details see Fig. S1 ^1^). The cellfree hydrogel used in condition A was still present at the end of culture and cell infiltration was absent. However, the hydrogel stained positive for GAGs (safranin-O) and collagen type II with a gradient in intensity. The highest staining intensity was observed at the defect bottom and sides and it faded towards the hydrogel surface. Similar results were already observed during a pilot study with plugs from a different donor (Fig. S2 ^1^).

In contrast to condition A, more intense staining for cartilaginous matrix components was detected throughout the defect area in condition B (Fig. 4). After 29 and 57 days of culture, homogeneously distributed cells and cell clusters were observed in the hydrogel. The hydrogel stained positive for GAGs (safranin-O) and collagen type II and negative for collagen type I. The pericellular matrix of cells within cell clusters stained positive for collagen type VI (Fig. S3 ^1^).

Defects filled with conditions C and D revealed a dense tissue layer at the bottom of the defect and at the cartilage-hydrogel interfaces, comparable to observations in condition A (Fig. 4, Fig. S3 ^1^). Additionally, cells and cell clusters were visible in the hydrogel with a homogeneous distribution, similar to the observations for condition B. Both the tissue layer and hydrogel stained strongly with safranin-O and the collagen type II antibody. In addition, the pericellular matrix of the cell clusters in the hydrogel and that of cells in the underlying dense tissue layer were respectively positive and negative for collagen type VI (Fig. S3 ^1^). No clear differences were observed between day 29 and day 57 samples (Fig. S4 ^1^). In addition, in a few samples of conditions B, C, and D, inhomogeneous remodeling was observed (Fig. S5 ^1^).

**Fig. 5:**
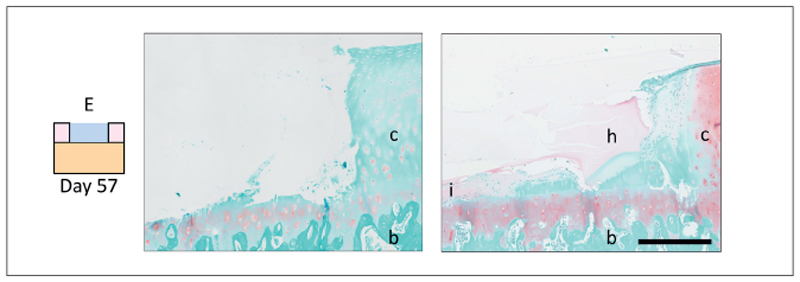
Cross-sectional overview of the side of the defect area of plugs filled with condition E No signs of tissue remodeling or repair were visible in most of the three samples (left, hydrogel absent) while in one sample, cells were visible at the bottom of the defect after 57 days of culture (right). Scale bar represents 400 μm; c, native cartilage; b, bone; I, cell infiltration; h, hydrogel.

After 57 days of culture, the defect area filled with condition E looked comparable to day 0 controls (Fig. 5). However, in one of the five samples, a thin cell layer formed at the bottom of the defect, below the hydrogel (Fig. 5). This cell layer stained positive for safranin-O and collagen type II; however, staining intensity was much lower compared to the repair tissue in the cell-laden conditions. Moreover, the location of this cell layer could not be linked to accidental damage in the calcified layer of the OC plug.

**Fig. 6:**
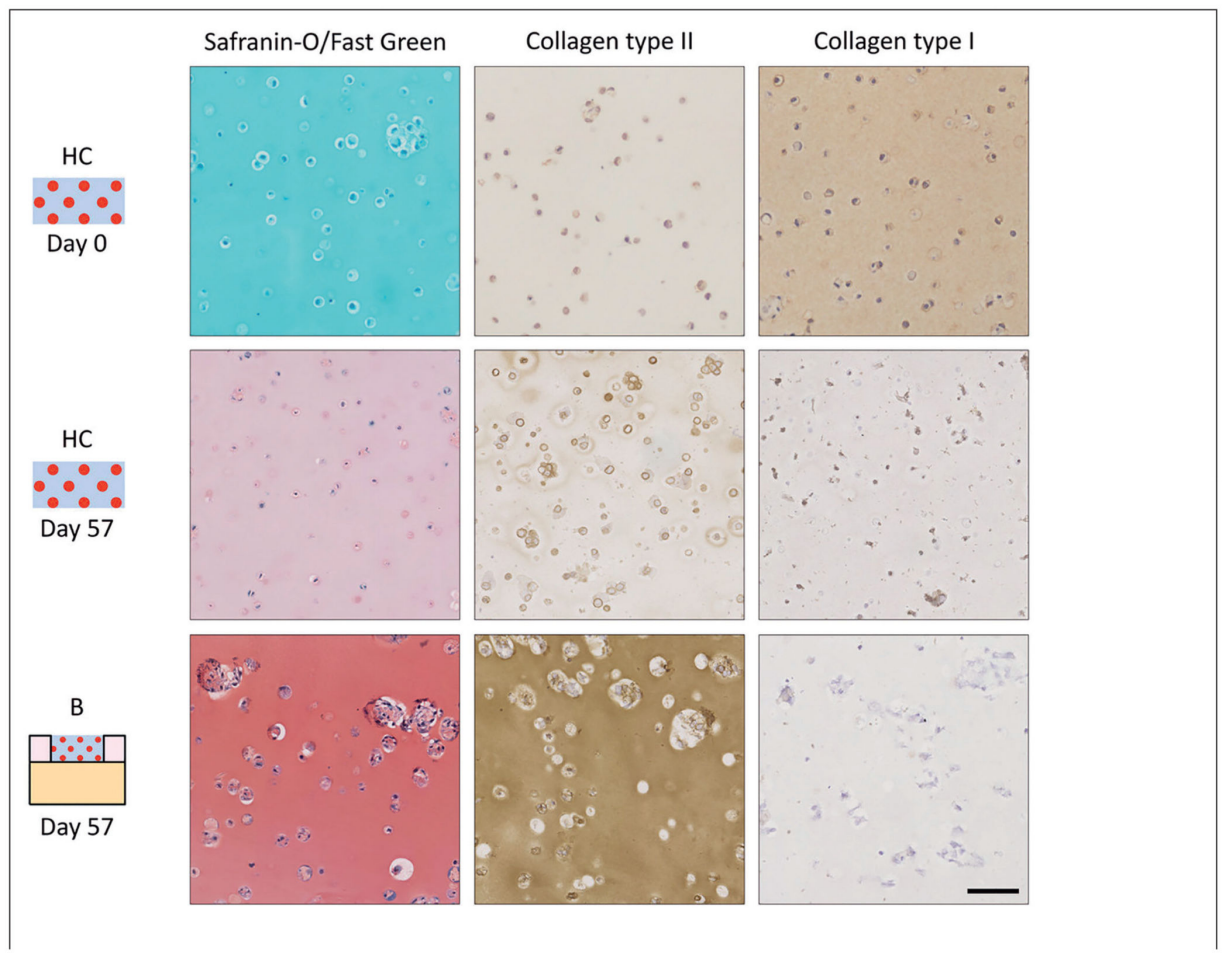
Overview of matrix production in hydrogel controls and the hydrogel constructs in condition B Scale bar represents 200 μm for all images; representative images of 4 plugs and 4 HCs.

HCs reacted strongly with the collagen type I antibody at day 0, but staining intensity decreased during culture (Fig. 6). At days 29 and 57, HCs stained positive with safranin-O and collagen type II, with highest staining intensities at day 57. Tissue formation in HCs differed from the tissue formation in the hydrogel constructs in condition B. Both safranin-O and collagen type II stainings were more intense in the constructs of condition B and contained larger cell clusters compared to HCs.

**Fig. 7:**
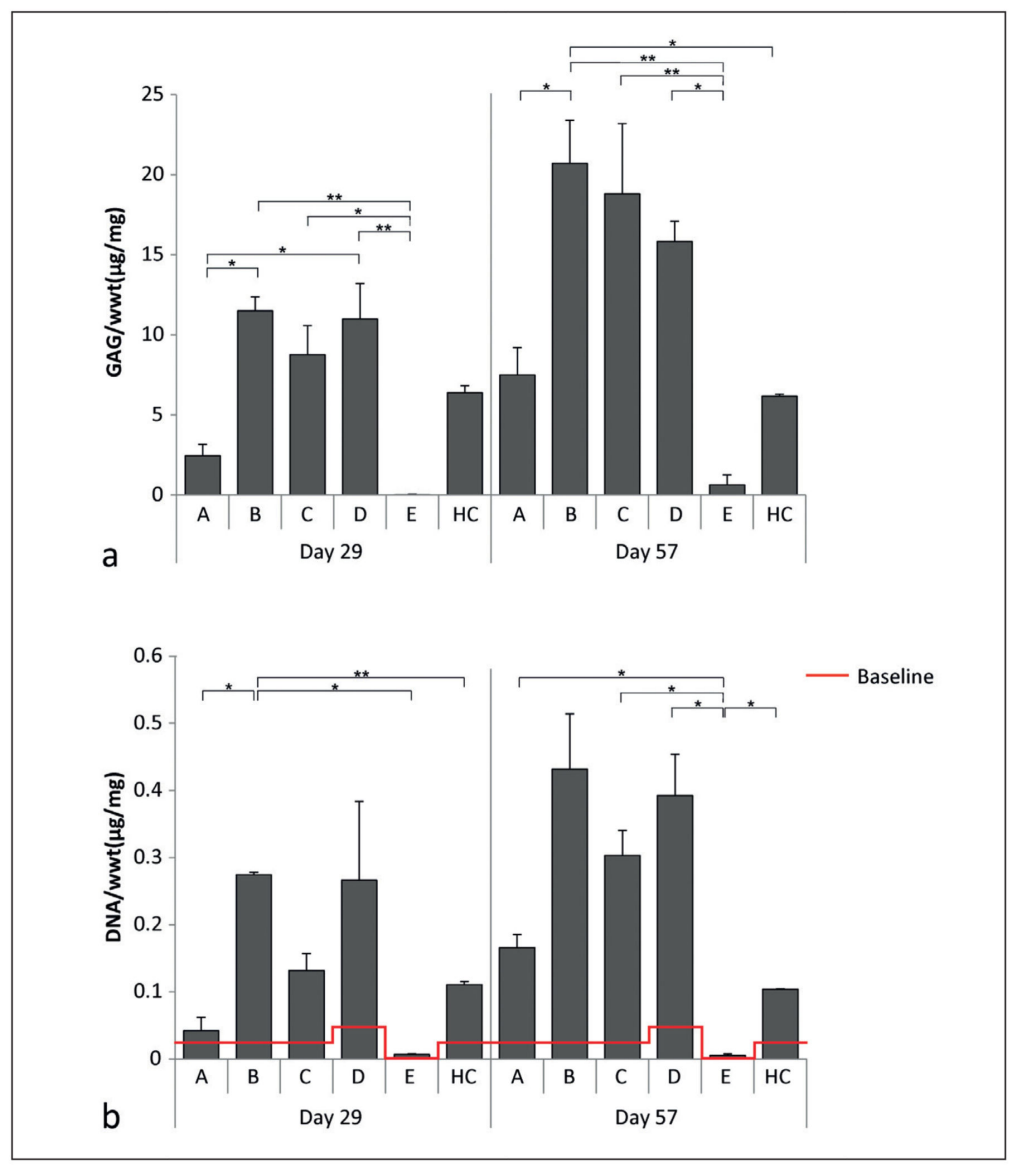
Quantitative measurements of the different defect fillings a) GAG normalized to wwt. No GAGs were detected in the defect filling at day 1. b) DNA normalized to the wwt. At day 1 (baseline, red), 0.023 ±0.0014 μg/mg DNA/wwt was detected for defects filled with conditions A, B, C, and HC, while 0.047 ±0.0029 μg/mg DNA/wwt was detected in plugs filled with condition D, and no DNA was detected for plugs filled with condition E. For condition A-D, 3 and 4 plugs were measured at days 29 and 57, respectively, while 2 and 3 plugs were measured respectively for condition E. *, p < 0.05; **, p < 0.01 between the connected conditions.

The amount of GAG/wwt was significantly higher in the newly formed tissue in conditions B and D compared to condition A at day 29 (Fig. 7a). At day 57, only the tissue in condition B contained significantly more GAG/wwt compared to A. No significant differences in GAG/wwt were observed between conditions C and D. At both time points, the GAG/wwt was significantly lower for the cell-free condition E compared to the conditions that had cells homogeneously encapsulated in the hydrogels (B-D).

The amount of DNA/wwt was significantly higher in newly formed tissue in condition B compared to conditions A, E and HO at day 29 (Fig. 7b). However, at day 57, significantly less DNA/wwt was present in the defect area of plugs with condition E compared to the conditions which had chondrocytes seeded at the defect bottom (A, C, and D) and compared to the HC.

### 3.3 Contribution of endogenous and delivered cells in cartilage regeneration

DNA profiles of the cells isolated from the repair tissue were identical to the DNA profiles of the chondrocyte donor (Tab. S1 ^1^). DNA of the plug donor was not detected in the repair tissue.

## 4 Discussion

This study confirms the potential of the *ex vivo* OC plug model for the evaluation and optimization of new hydrogel-based cartilage repair strategies. The current study focused on equine OC plugs, as the horse is a widely used pre-clinical model for cartilage repair strategies (Malda et al., 2012). Changes in the cartilage during culture observed for the equine plugs were similar to those reported for other species (Chen et al., 1997; de Vries-van Melle et al., 2012; Elson et al., 2015; Schwab et al., 2016). The native cartilage swelled and GAGs leached into the medium, possibly due to the damaged collagen network at the surface of the plugs and the defect edges (Chen et al., 1997; de Vries-van Melle et al., 2012; Elson et al., 2015; Schwab et al., 2016). However, in the current study, both the water and GAG content were stable between days 29 and 57, even though GAGs leached continuously into the medium, indicating that GAGs were produced and that a new stable situation was reached.

Different variations of hydrogels were cultured within the cartilage defects of the OC plugs and this resulted in the formation of hyaline-like cartilage tissue. Surprisingly, culture of cell-laden hydrogels in the OC plug improved matrix deposition by the delivered chondrocytes. Significantly higher DNA/wwt and GAG/ wwt contents were observed in the cartilage defects of plugs filled with gels from condition B, compared to the HCs, which contained the same chondrocyte density. Additionally, the hydrogels in condition B stained more intensely for GAGs and collagen type II and contained larger cell clusters compared to the HCs. GAG and DNA levels of the HC samples are in line with previous findings (Mouser et al., 2016b). The hydrogel in condition B was thinner compared to HC constructs (~1 mm and 2 mm, respectively), which might result in differences in the availability of nutrients. However, HCs were cultured while floating in the medium and the diffusion distance to reach the core of the samples was much smaller than the maximal distance for sufficient diffusion of nutrients (Griffith et al., 2005). Therefore, the increase in proliferation and matrix production in the hydrogel of condition B is expected to be the result of culturing the cell-laden hydrogels within cartilage defects, suggesting that the cells within the OC plug secrete factors that influence the delivered chondrocytes (Sherwood et al., 2014). Indeed, it has been demonstrated before that chondrocytes release factors that stimulate chondrocyte proliferation, matrix synthesis, and remodeling when cartilage damage occurs, i.e., fibroblast growth factor, TGF-β, and bone morphogenetic proteins (Dell’Accio et al., 2008; Ellsworth et al., 2002; Sherwood et al., 2014; Vincent et al., 2002).

These observations highlight the potential of the *ex vivo* OC plug model for the replacement of animal models during the evaluation and optimization phase of new cartilage repair strategies. In general, new cartilage repair constructs are tested subcutaneously or intramuscularly in rodent models for safety and mechanistic studies, followed by implantation at orthotopic locations in larger animal models, e.g., lapine, canine, porcine or equine, to evaluate construct functioning and cartilage repair (Chu et al., 2010). The *ex vivo* OC plug model may refine larger animal studies and even reduce the number of necessary animals, as it allows optimization of basic construct properties, e.g., the cell distribution, using slaughterhouse waste material. As such, it allows a more accurate screening system to identify the optimal, most promising therapies, therefore reducing the number of experimental groups to be tested *in vivo*. Nevertheless, although the orthotopic-like environment provided by the *ex vivo* OC plug model is promising, it remains a simplification of the native environment of the whole joint. Therefore, validation of our findings in larger animal models is still required to determine the extent to which the *ex vivo* OC plug model can predict clinical outcomes. Moreover, the possibility of studying long-term tissue repair with the OC plug model has yet to be determined and validated with the *in vivo* response. Such comparison would help evaluate the potential of the plug model to further reduce the need for *in vivo* experiments.

The results of this study confirm the therapeutic potential of chondrocyte-laden hydrogels for cartilage repair and demonstrate a crucial role of the initial spatial chondrocyte distribution in the hydrogel for tissue repair. More specifically, homogeneous chondrocyte encapsulation in gelMA/gellan hydrogels is beneficial for relatively fast defect filling with new cartilage-like tissue, while seeding the chondrocytes at the defect bottom improves local construct integration. When chondrocytes were positioned at the bottom of the defect, enclosed by a cell-free hydrogel (condition A), a dense cartilage-like tissue layer, rich in GAGs and collagen type II, formed on the calcified layer. Nevertheless, after 57 days of culture, the defect was not completely filled with tissue. When encapsulating the same number of chondrocytes homogeneously in the hydrogel (condition B), differentiating cell clusters rich in GAGs and collagen type II formed. The formation of such clusters is often observed in cartilage tissue engineering using cell-laden hydrogel constructs, and was also reported for gelMA-based constructs after 2 months of subcutaneous implantation in a rat model (Boere et al., 2015). For a complete mimicry of cartilage architecture, these clusters should mature and reorganize according to the zone in which they are located; however this will depend also on the degradation profile of the hydrogel and its longer-term performance, which could be the subject of future studies. Similarly, the evolution of the mechanical properties of the hydrogel should also be evaluated in future studies to fully explore the potential of gelMA/gellan hydrogels for cartilage regeneration. Furthermore, plugs filled with condition B contained neo-tissue, which was distributed throughout the entire defect after 57 days of culture. Although condition A resulted in less cartilage-like tissue and chondrocytes adopted a more fibroblast-like phenotype (lack of chondron formation and increase in collagen type I compared to homogeneously encapsulated chondrocytes), the tissue that had formed filled all irregularities at the bottom of the defect, while the newly formed tissue in condition B revealed an abrupt transition. When filling the defect with a combination of both methods (conditions C and D), the best results were obtained: Both a dense tissue layer developed at the defect bottom and differentiating cell clusters formed in the hydrogel. This was independent of the initial cell density.

Significantly more DNA/wwt and GAG/wwt was measured in the repair tissue of condition B compared to A. To ensure proper chondrocyte engraftment at the defect bottom, OC plugs with seeded chondrocytes were incubated for a total of three hours, exceeding the attachment time recommended by Chen et al. (1997). Additionally, no cells were detected in the incubation medium when it was refreshed in the present study, confirming effective cell delivery. The lower DNA/wwt values at day 29 in condition A are, therefore, probably the result of less proliferation compared to conditions B-D. Due to the relatively high cell density at the defect bottom in condition A, the chondrocytes have limited space to proliferate. Additionally, absence of cell-cell contact is known to stimulate chondrocyte proliferation, which also explains the formation of cell clusters in the hydrogels with homogeneous cell encapsulation (Abbott and Holtzer, 1966). Moreover, in pellet cultures where chondrocyte densities are relatively high, limited chondron formation is observed (Stewart et al., 2000), while chondrons are more active in cartilage-like matrix production compared to chondrocytes without their pericellular matrix (Zhang, 2015). Indeed, the pericellular matrix of the cells encapsulated in the hydrogel stained positive for collagen type VI, while this was not the case for the cells in the dense tissue layer, highlighting the potential of hydrogels with encapsulated chondrocytes for cartilage repair.

Increasing the total number of delivered chondrocytes did not result in increased tissue formation or DNA levels (condition C compared to D). This observation is in line with other studies that demonstrated that differences in DNA levels and tissue formation due to different initial cell densities disappear during long-term culture (Hansen et al., 2013; Kon et al., 2010). However, other studies reported beneficial effects of increased cell densities using higher initial cell densities compared to the current study (Chen et al., 1997; Puelacher et al., 1994; Talukdar et al., 2011). Possibly, the effect of different cell densities is influenced by the initial cell density and the scaffold/hydrogel in which the chondrocytes are grown.

The findings of this study may impact on cell-based cartilage repair strategies currently used in the clinic, as the majority of these do not provide homogeneous spatially distributed chondrocytes (Huang et al., 2016). ACI involves cell seeding in the defect depth and scaffold-based approaches, e.g., NOVOCART 3D^®^, Bioseed^®^, or MACI, are often associated with limited penetration of seeded chondrocytes into the scaffold (Huang et al., 2016). Although scaffold-based approaches show promising results, incomplete and moderate defect filling was reported in patients treated with NOVOCART 3D^®^ (Niethammer et al., 2016) or Bioseed^®^ (Kreuz et al., 2011) and MACI (Meyerkort et al., 2014), respectively. Chondrocyte penetration into a scaffold can be improved by pre-culturing the cell-laden scaffolds in perfusion bioreactors (Pei et al., 2002). However, hydrogels are ideal candidates for the generation of constructs with homogeneously encapsulated chondrocytes without requiring bioreactors.

When seeding cells in the defects, tissue outgrowth originating from the delivered cells was observed in the present study. Similar effects were observed in *in vitro* cultures of collagen type I/II patches for MACI (Russlies et al., 2002). This tissue outgrowth might be prevented by incorporating a thin dense polymer layer at the hydrogel surface facing the joint space, as demonstrated to be effective for MACI (Brittberg, 2010; Huang et al., 2016) and NOVOCART 3D^®^ (Huang et al., 2016) scaffolds. Three-dimensional printing techniques may facilitate the development of cell-laden hydrogel constructs with such a “sealed” surface, as well as more complex chondrocyte delivery approaches, e.g., depth-dependent differences, to further resemble the zonal architecture of native cartilage (Mouser et al., 2016a).

Cell delivery was required to obtain repair tissue in the defect. No tissue was formed in plugs filled with cell-free hydrogels (condition E) and repair tissue was solely formed by the delivered cells as determined by genetic profiling, confirming the potential of cell implantation for cartilage repair. However, chondrocytes from the native cartilage can migrate into the defect area, as demonstrated with cell-free collagen type I hydrogels (Gavenis et al., 2010). However, these hydrogels likely contained different polymer and cross-linking densities favoring migration and were fixated with chemo-attractive fibrin glue (Kirilak et al., 2006), which could explain the different observation compared to our study (Kirilak et al., 2006). Further research is required to determine the optimal cell concentration for the treatment of cartilage defects with gelMA/gellan hydrogels.

Although complete defect filling could be reached in two months with the delivery of cell-laden gelMA/gellan (conditions B, C, and D), none of the cell-based strategies resulted in the restoration of the depth-dependent organization of articular cartilage. To accomplish this, other strategies should be implemented such as the incorporation of zonally harvested chondrocytes or zonal distribution of tissue-inductive cues (Fujie et al., 2015; Schuurman et al., 2015). Another important next step is the incorporation of mechanical stimulation, which can increase matrix production of delivered chondrocytes and is believed to contribute to the organization of collagen fibers (Benninghoff, 1925; Bian et al., 2010; Kock et al., 2013; Mauck et al., 2002).

## 5 Conclusions

The *ex vivo* OC plug model forms a promising tool for the evaluation and optimization of new hydrogel-based cartilage repair strategies and therefore may reduce the need for animal experiments. Moreover, the *ex vivo* OC plug model stimulates cartilage-like tissue formation by cells delivered with gelMA/ gellan hydrogel in full-thickness cartilage defects. The morphology and quantity of the repair tissue is dominated by the initial spatial chondrocyte distribution. Seeding cells at the defect bottom and encapsulating them in the hydrogel resulted in a well-integrated repair tissue that completely filled the defect after two months. Repair tissue was formed by the delivered chondrocytes, confirming the potential of cell delivery for cartilage repair. Overall, these findings demonstrate the important role of spatial chondrocyte distribution in hydrogel constructs on cartilage tissue formation. Hence, this study may impact future cell delivery approaches for cartilage repair.

## Supplementary Material

Supplementary figures and table

## Footnotes

1 doi:10.14573/altex.1704171s
