# Supplementary material for: Ex Vivo Model Unravelling Cell Distribution Effect in Hydrogels for Cartilage Repair
Source: ALTEX. Author manuscript; Available in PMC 2020 Oct 16. (PMC7116182; doi:10.14573/altex.1704171)
Supplement: Supplementary figures and table [file EMS96596-supplement-Supplementary_figures_and_table.pdf]

Mouser et al.:

Ex vivo Model Unravelling Cell Distribution Effect in Hydrogels for Cartilage Repair

Supplementary Data

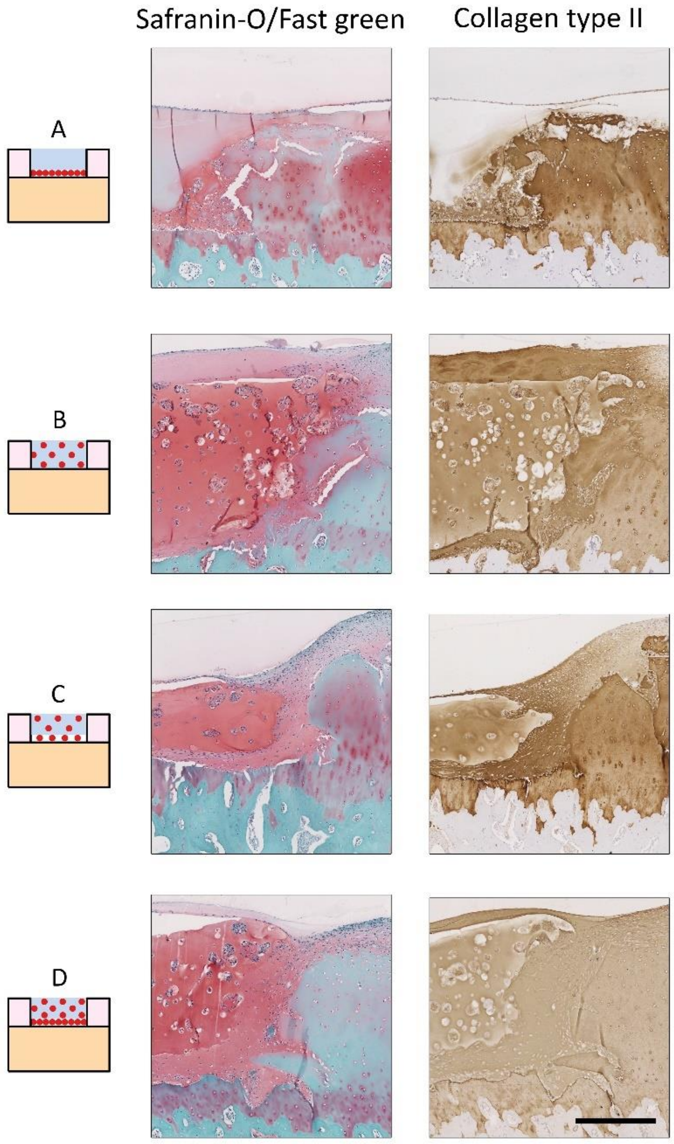

Fig. S1: View of the cartilage-hydrogel interface for plugs filled with each condition (A-D as defined in Fig. 1)
Scale bar represents 400 µm and is the same for all histological images.

This is an Open Access article distributed under the terms of the Creative Commons Attribution 4.0 International license (http://creativecommons.org/licenses/by/4.0/), which permits unrestricted use, distribution and reproduction in any medium, provided the original work is appropriately cited.

doi:10.14573/altex.1704171s

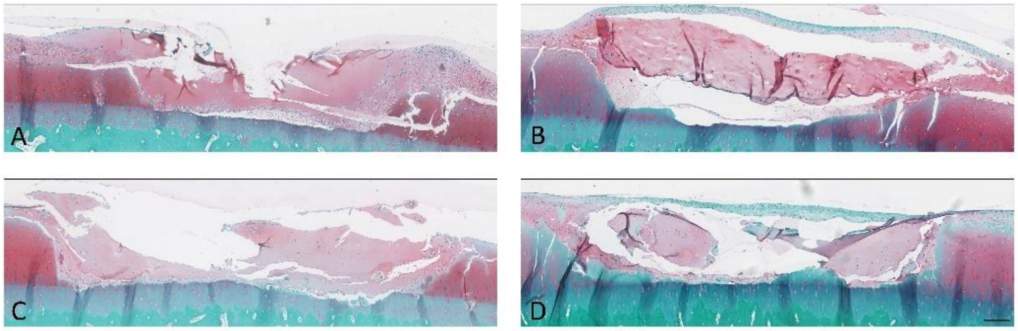

Fig. S2: Cross-sectional overview of a safranin-O/Fast green staining of the cartilage defects filled with conditions A-D (as defined in Fig. 1) for a second plug donor (# plugs = 3 for each condition) at day 57
Scale bar represents 400 µm and is the same for all histological images.

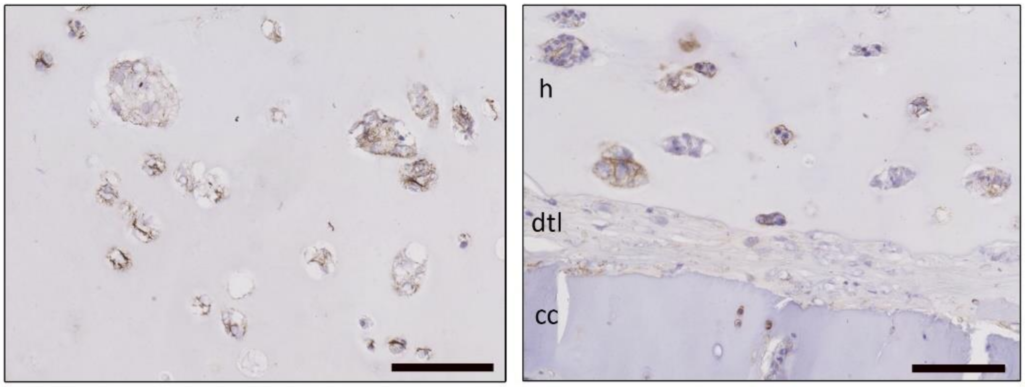

Fig. S3: Collagen type VI staining of the hydrogel in a defect of condition B (left, # plugs = 4) and of a defect cross-section of condition C (right, # plugs = 4) at day 57
Scale bars represent 100 µm; h, hydrogel; dtl, dense tissue layer; cc; calcified cartilage.

Interpretation: The pericellular matrix of the cells in the hydrogel stained positive for collagen type VI, while the pericellular matrix of the chondrocytes in the dense tissue layer was negative for collagen type VI.

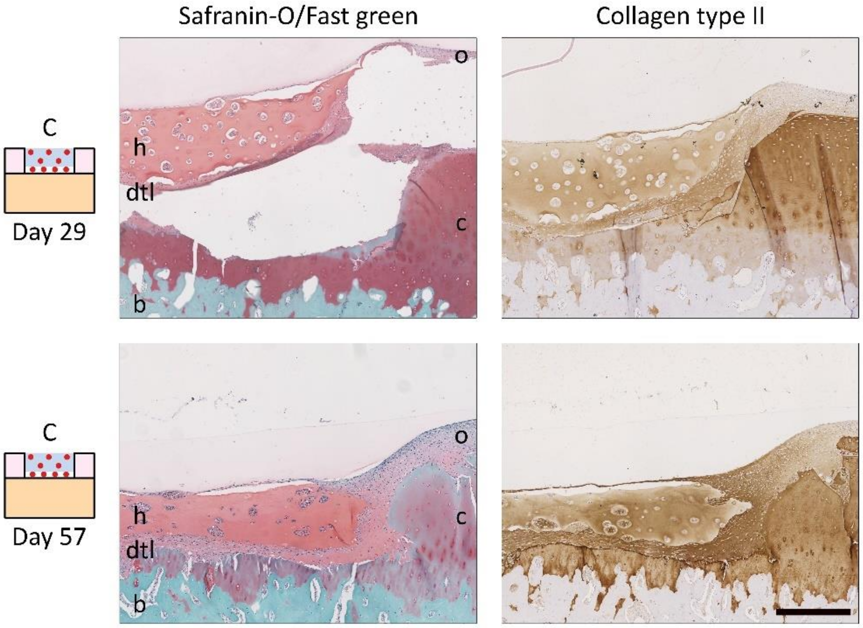

Fig. S4: Cross-sectional overview of the defect sites of condition C at days 29 (# plugs = 3) and 57 (# plugs = 4)
In the safranin-O images, c, native cartilage; b, bone; h, hydrogel; o, tissue outgrowth; dtl, dense tissue layer.

Interpretation: No clear differences were observed between days 29 and 57 for each condition. The dense tissue layer observed at the defect bottom in conditions A, C, and D did not thicken over time and the size of cell clusters observed in conditions B, C, and D did not change.

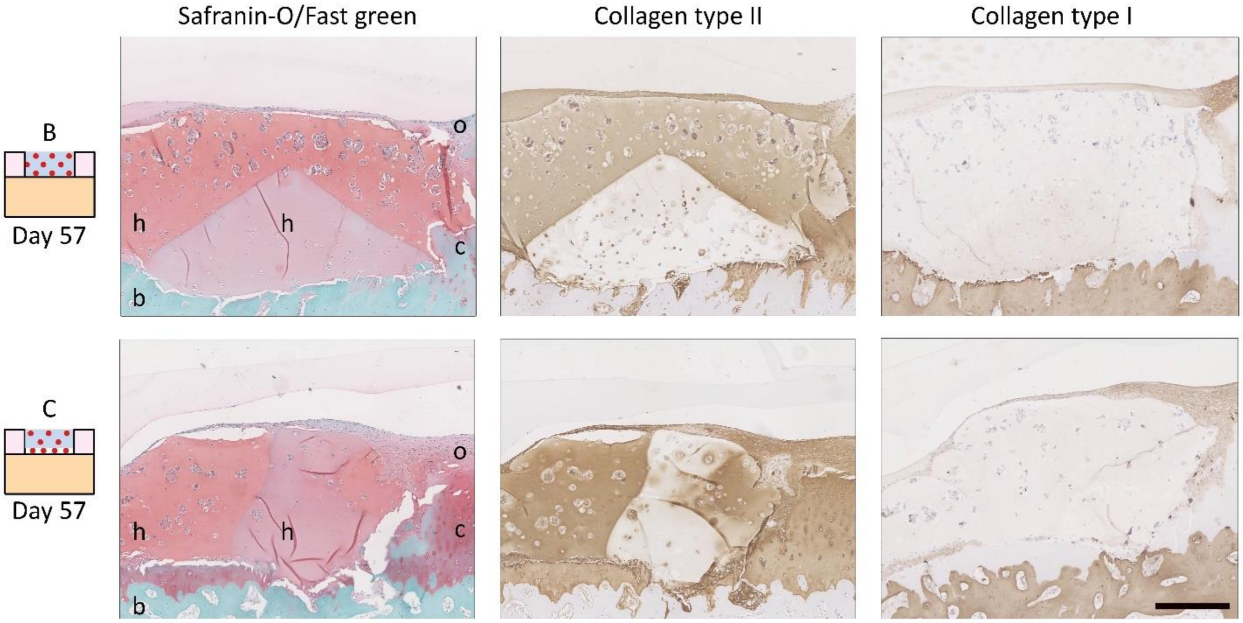

Fig. S5: Examples of inhomogeneous matrix formation in the xy-plane of defects in conditions B (top) and C (bottom) at day 57.
Scale bar represents 400 µm; c, native cartilage; b, bone; h, hydrogel; o, tissue outgrowth.

Interpretation: Inhomogeneous tissue remodeling was observed in a few samples of conditions B, C, or D. These samples showed a sharp transition in the cell-laden hydrogel from an area with relatively large cell clusters and intense safranin-O and collagen type II staining to an area with smaller cell clusters and less intense safranin-O and collagen type II staining. In addition, collagen type I staining was more intense in the areas containing the lower collagen type II and safranin-O staining compared to the other area. The inhomogeneous remodeling, resulting in areas with relatively high or low cartilage-like tissue formation, is likely related to inhomogeneous degradation of gelMA/gellan hydrogels. This hypothesis is supported by the collagen type I staining. As gelMA is generated from denatured collagens, mainly type I, it stains positive for collagen type I at the beginning of culture. However, after the culture period, the collagen type I positive signal of the hydrogel diminished (Fig. 5, main text), indicating degradation of the hydrogel. Contrarily, the tissue areas containing relatively low cartilage-like tissue were still homogeneously positive for collagen type I at day 57. A possible explanation for this observation could be limitations in diffusion of degrading or remodeling factors secreted by the cells of the OC plug or of the TGF-β supplemented in the medium.

Tab. S1: DNA profiles of the OC plug donor, chondrocyte donor, and the repair tissue growing out of the defect area

DNA marker	ISAG code*		
	**OC plug donor**	**Chondrocyte donor**	**Samples**
AHT4	H / J	O / -	O / -
AHT5	N / -	J / K	J / K
HMS1	M / -	I / M	I / M
HMS2	M / -	K / R	K / R
HMS6	L / O	M / P	M / P
HMS7	L / O	L /N	L /N
HTG4	L / -	K / M	K / M
HTG6	J / -	G / -	G / -
HTG7	M / N	M / O	M / O
VHL20	I / -	I / N	I / N
ASB2	- / -	K / N	K / N
HMS3	I / M	P / -	P / -
HTG10	M / O	I / O	I / O
ASB17	N / R	M / O	M / O
ASB23	I / K	K / -	K / -
LEX3	N / -	P / -	P / -
CA425	M / N	J / -	J / -

*ISAG = International Society of Animal Genetics

Interpretation: The DNA profile of the tissue growing out of the defect area (# plugs = 4) was identical to the DNA profile of the chondrocyte donor, while no DNA of the OC plug donor was present in the outgrowth tissue.
